# Detection of Subclinical Cardiovascular Disease by Cardiovascular Magnetic Resonance in Lymphoma Survivors

**DOI:** 10.1016/j.jaccao.2021.09.015

**Published:** 2021-12-21

**Authors:** Nikki van der Velde, Cécile P.M. Janus, Daniel J. Bowen, H. Carlijne Hassing, Isabella Kardys, Flora E. van Leeuwen, Cynthia So-Osman, Remi A. Nout, Olivier C. Manintveld, Alexander Hirsch

**Affiliations:** a Department of Cardiology, Thoraxcenter, Erasmus Medical Center, University Medical Center Rotterdam, Rotterdam, the Netherlands; b Department of Radiology and Nuclear Medicine, Erasmus Medical Center, University Medical Center Rotterdam, Rotterdam, the Netherlands; c Department of Radiation Oncology, Erasmus Medical Center Cancer Institute, Rotterdam, the Netherlands; d Department of Psychosocial Research and Epidemiology, Netherlands Cancer Institute, Amsterdam, the Netherlands; e Department of Hematology, Erasmus Medical Center, University Medical Center Rotterdam, Rotterdam, the Netherlands

**Keywords:** cardiac function, cardiotoxicity, cardiovascular magnetic resonance, Hodgkin lymphoma, myocardial strain, CMR, cardiovascular magnetic resonance, CVD, cardiovascular disease, GCS, global circumferential strain, GLS, global longitudinal strain, HL, Hodgkin lymphoma, LGE, late gadolinium enhancement, LV, left ventricular, NHL, non-Hodgkin lymphoma

## Abstract

**Background:**

Long-term survivors of Hodgkin lymphoma (HL) and mediastinal non-Hodgkin lymphoma experience late adverse effects of radiotherapy and/or anthracycline-containing chemotherapy, leading to premature cardiovascular morbidity and mortality.

**Objectives:**

The aim of this study was to identify markers for subclinical cardiovascular disease using cardiovascular magnetic resonance (CMR) in survivors of HL and non-Hodgkin lymphoma.

**Methods:**

CMR was performed in 80 lymphoma survivors treated with mediastinal radiotherapy with or without anthracyclines, and results were compared with those among 40 healthy control subjects matched for age and sex.

**Results:**

Of the 80 lymphoma survivors, 98% had histories of HL, the mean age was 47 ± 11 years, and 54% were male. Median radiotherapy dose was 36 Gy (interquartile range: 36-40 Gy), and radiotherapy was combined with anthracyclines in 70 lymphoma survivors (88%). Mean time between diagnosis and CMR was 20 ± 8 years. Significantly lower left ventricular (LV) ejection fraction (53% ± 5% vs 60% ± 5%; *P* < 0.001) and LV mass (47 ± 10 g/m^2^ vs 56 ± 8 g/m^2^; *P* < 0.001) and higher LV end-systolic volume (37 ± 8 mL/m^2^ vs 33 ± 7 mL/m^2^; *P* = 0.013) were found in lymphoma survivors. LV global strain parameters were also significantly worse in lymphoma survivors (*P* < 0.02 for all). Native myocardial T1 was significantly higher in lymphoma survivors compared with healthy control subjects (980 ± 33 ms vs 964 ± 25 ms; *P* = 0.007), and late gadolinium enhancement was present in 11% of the survivors.

**Conclusions:**

Long-term lymphoma survivors have detectable changes in LV function and native myocardial T1 on CMR. Further longitudinal studies are needed to assess the implication of these changes in relation to treatment and clinical outcome.

In recent decades, improvements in the treatment of patients with Hodgkin lymphoma (HL) and non-Hodgkin lymphoma (NHL) have resulted in an increased survival rate of approximately 80% to 90%. Nevertheless, long-term survivors may experience several late adverse effects that may lead to premature morbidity and mortality (1, 2, 3). Cardiovascular diseases (CVDs), such as myocardial dysfunction, heart failure, myocardial infarction, and valvular disease, are among the most important adverse effects of mediastinal radiotherapy and/or anthracycline-containing chemotherapy, which can develop even decades after treatment initiation (1,4, 5, 6). However, lymphoma survivors may have CVD without having clinical symptoms, and normal left ventricular (LV) ejection fraction does not preclude the presence of myocardial dysfunction (7,8). To detect early stages of late cardiotoxicity, screening for subclinical CVD through appropriate monitoring, including noninvasive cardiac imaging on transthoracic echocardiography or cardiovascular magnetic resonance (CMR), should be considered (1,9). Both transthoracic echocardiography and CMR are safe, nonionizing imaging modalities that are able to assess myocardial and valvular function. In addition, CMR has the unique ability to characterize tissue. Late gadolinium enhancement (LGE) imaging and mapping techniques enable the detection of (diffuse) myocardial fibrosis and/or edema (1,9,10). Limited data exist on CMR abnormalities associated with subclinical CVD in survivors of HL and NHL (5). Therefore, in this study we aimed to identify markers of subclinical CVD using CMR in survivors of HL and NHL.

## Methods

### Study population and healthy control subjects

For this single-center cross-sectional study, we screened lymphoma survivors of the BETER (Better Care After [Non]-HL, Evaluation of Long-Term Treatment Effects and Screening Recommendations) outpatient clinic of the Erasmus Medical Center between February 2018 and October 2020 (11). Lymphoma survivors with medical histories of HL or mediastinal NHL treated with mediastinal radiotherapy with or without anthracyclines were eligible for inclusion if they were ≥18 years of age and at least 5 years free of disease. Definitions regarding stage and grade of HL are given in the Supplemental Methods. Exclusion criteria were treatment for secondary malignant disease, contraindications to CMR (ie, claustrophobia), and a known history of surgical or percutaneous intervention for valvular and/or coronary artery disease. None of the included lymphoma survivors had a known history of coronary artery disease. In all patients, 12-lead electrocardiogram, transthoracic echocardiography, and CMR were performed. The lymphoma survivors were compared with healthy control subjects, matched for age and sex on a group level and without CVD, who underwent CMR examination between June 2018 and November 2019. This study is part of the PROCARBI (Prospective, Explorative Cohort Study to Correlate Cardiac Biomarkers With Late Cardiotoxicity Induced by Radiotherapy Alone or Combined With Anthracyclines for HL) study (NL7958). This study was approved by the Institutional Review Board, and written informed consent was obtained from all patients and healthy control subjects (MEC17-505/MEC-2014-096).

### Electrocardiography and echocardiography

Standard 12-lead electrocardiography was performed at rest and was scored for heart rate, rhythm, conduction times, T-wave amplitude, and ST-segment deviations. In case of prolongation of the QRS complex (>120 ms), the type of bundle branch block was noted.

Transthoracic echocardiography was performed on a Philips Epiq 7C using a standardized acquisition protocol, and parameters related to diastolic function and valvular disease were measured. LV diastolic function was defined as normal, grade I (abnormal relaxation), grade II (pseudonormal), grade III (restrictive filling), or unclear on the basis of Doppler mitral inflow pattern parameters, including early (E) and late (A) LV filling velocities, E/A ratio, and tissue Doppler imaging–derived septal early diastolic velocities (e′) (12). Valvular disease was scored as none, mild, moderate, or severe according to the same recommendations.

Electrocardiography and transthoracic echocardiography were not performed in healthy control subjects.

### Cardiovascular magnetic resonance

CMR examinations were performed on a SIGNA Artist 1.5-T scanner (GE Healthcare) with a dedicated anterior-array coil, electrocardiographic gating, and breath-hold techniques. The detailed scan protocol and scan parameters are described in the Supplemental Methods.

Steady-state free precession cine images were obtained during breath-hold in all long-axis views (2-, 3-, and 4-chamber) and in a contiguous short-axis stack, with coverage from base to apex. LV and right ventricular dimensions, systolic function, and LV mass were determined on the short-axis images. Volumes and mass were corrected for body surface area.

Strain analyses using 2-dimensional feature tracking CMR were performed. Myocardial LV global longitudinal strain (GLS) was measured using all long-axis views. Endocardial right ventricular GLS was measured in the 4-chamber view. Myocardial global circumferential strain (GCS) and myocardial global radial strain measurements were performed using a basal, midventricular, and apical short-axis view. In addition, systolic and diastolic hemodynamic forces were calculated per entire heartbeat.

Images for T1 mapping were obtained in a midventricular short-axis slice, using a modified Look-Locker inverse recovery sequence with a 5(3)3 acquisition scheme pre-contrast and a 4(1)3(1)2 acquisition scheme post-contrast. The same slice location was used for T2 mapping. The whole myocardium in the midventricular slice was included. Only in the case of artifacts was part of the myocardium excluded. Motion correction was performed. A blood sample was collected immediately after CMR examination for the determination of hematocrit in order to calculate extracellular volume fraction. The cell volume was calculated using the following formula: LV mass (indexed)/1.05 × [1 − extracellular volume] (13). Phase-sensitive LGE imaging was performed at least 10 to 15 minutes after intravenous administration of a gadolinium-based contrast agent (0.2 mmol/kg; Gadovist), using a breath-held 2-dimensional segmented inversion-recovery gradient-echo pulse sequence. Images were obtained in all long-axis views and short-axis views. If necessary, the preset inversion time was adjusted to null normal myocardium for LGE imaging. LGE was visually scored as presence or absence, and if applicable the pattern and localization were assessed. In patients with LGE, the percentage LGE of the LV was measured using the 4-SD thresholding quantification technique.

Dedicated software was used for these measurements (Qmass version 8.1, Qstrain version 2.0.82.6, and Qmap T1/T2 version 2.2.38, Medis Medical Imaging).

### Clinical assessment

Baseline was defined as the date of CMR. The collected patient data included general, cardiovascular, and oncologic data; physical examination; and laboratory values. In addition, lymphoma survivors were stratified according to: 1) total mediastinal radiotherapy dose ≤36 and >36 Gy (median); and 2) treatment with or without high-dose anthracyclines. High-dose anthracyclines were defined as doxorubicin ≥300 mg/m^2^, epirubicin ≥540 mg/m^2^, or mitoxantrone ≥90 mg/m^2^.

### Statistical analysis

Continuous data were tested for normality before analysis using the Kolmogorov-Smirnov test and are expressed as mean ± SD or median (interquartile range), as appropriate. Categorical variables are presented as number (percentage).

Baseline characteristics were compared between lymphoma survivors and healthy control subjects, with aspects of CVD described in survivors. Subsequently, survivors were stratified according to radiotherapy and anthracycline dose for the comparison of baseline, transthoracic echocardiographic, and CMR characteristics. Continuous variables were compared using Student’s *t*-test or the Mann-Whitney *U* test depending on their distributions, and categorical data were compared using the Pearson chi-square test or the Fisher exact test, as appropriate.

Univariable and multivariable linear regression analyses were used to assess the associations of baseline and treatment variables with LV ejection fraction, GLS, GCS, and LV mass in lymphoma survivors. All variables with *P* values <0.20 in the univariable linear regression were included in a multivariable analysis (multivariable model 1). A second model was performed adding the following variables irrespective of *P* value: duration between diagnosis and CMR and total dose of anthracycline chemotherapy (multivariable model 2). Results are presented as parameter estimated (β) and 95% CI. Multicollinearity was assessed using the variance inflation factor.

All analyses were 2-tailed, and *P* values <0.05 were considered to indicate statistical significance. Statistical analyses were performed using SPSS version 25 (IBM).

## Results

**Figure 1:**
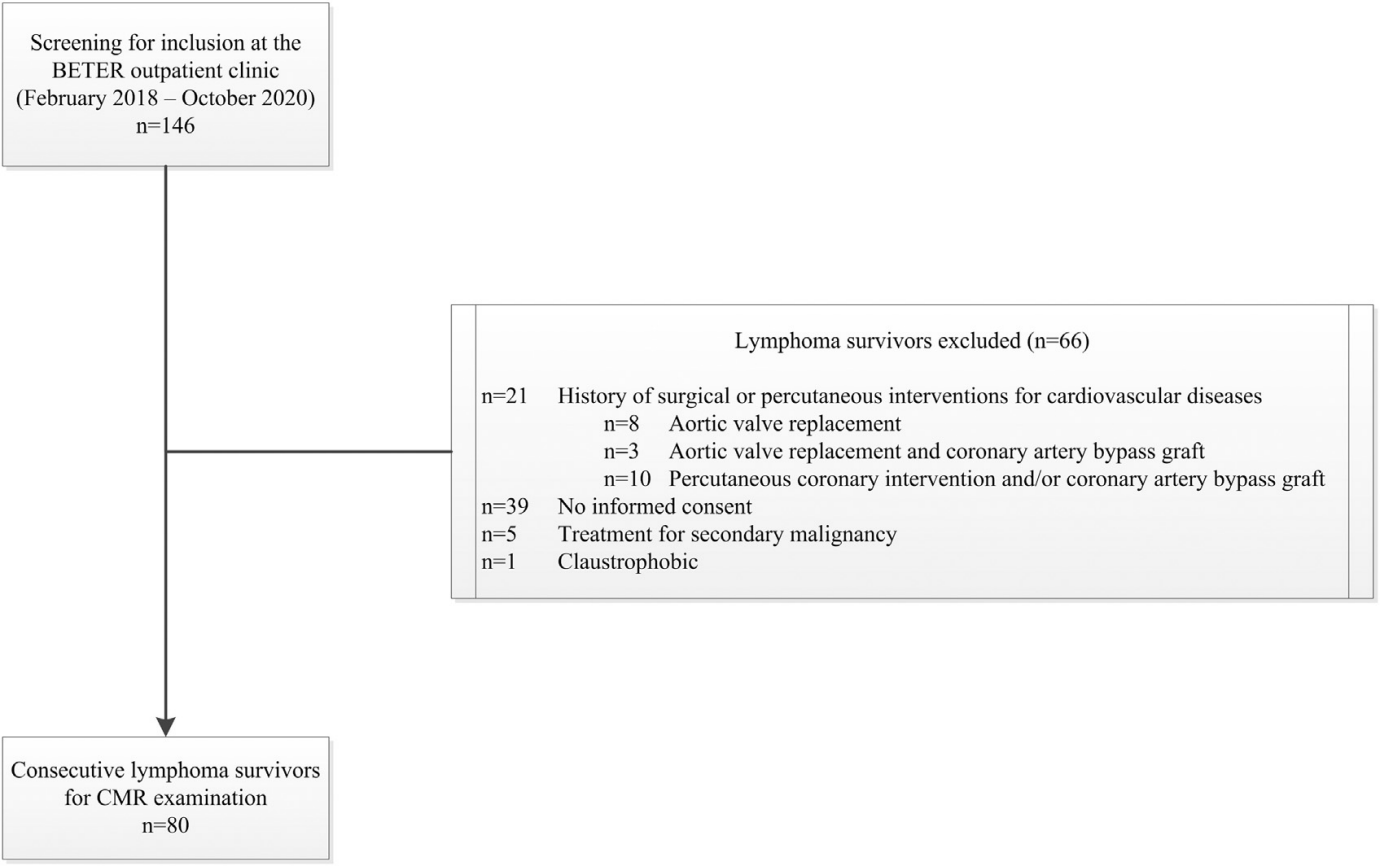
Flowchart of Patient Selection A total of 80 consecutive (non-)Hodgkin lymphoma survivors were included in this study and underwent cardiovascular magnetic resonance (CMR) examination. BETER = Better Care After (Non)-Hodgkin Lymphoma, Evaluation of Long-Term Treatment Effects and Screening Recommendations.

A total of 80 consecutive HL (n = 78) and mediastinal NHL (n = 2) survivors and 40 healthy control subjects matched for age and sex on a group level were included in this study (Figure 1). Baseline characteristics of both groups are summarized in Table 1. Overall, the mean age was 47 ± 11 years, and 53% were male.

**Table 1:** Baseline Characteristics of (Non)-Hodgkin Lymphoma Survivors Compared With Healthy Control Subjects

	Healthy Control Subjects (n = 40)	Lymphoma Survivors (n = 80)	*P* Value
Demographics			
Age at CMR (y)	47 ± 11	47 ± 11	0.92
Male	21 (53)	43 (54)	0.90
Body mass index (kg/m^2^)	24 (22-25)	25 (22-29)	0.065
Heart rate (beats/min)	58 (54-64)	76 (78-84)	<0.001
Systolic blood pressure (mm Hg)		130 ± 17	
Diastolic blood pressure (mm Hg)		81 ± 10	
Diagnosis			
Hodgkin		78 (98)	
Mediastinal non-Hodgkin		2 (3)	
Duration between diagnosis and CMR (y)		20 ± 8	
Stage			
I		8 (10)	
II		59 (74)	
III		12 (15)	
IV		1 (1)	
Grade^a^			
Favorable		24 (30)	
Unfavorable		38 (48)	
Unknown		18 (23)	
Previous therapy for lymphomas			
First chemotherapy treatment		72 (90)	
First chemotherapy regimen			
AVBD		32 (40)	
AVBD + DHAP + MOPP		1 (1)	
BEACOPP		2 (3)	
CHOP		1 (1)	
CHOP + MOPP/AVB(D)		2 (3)	
EVBP		8 (10)	
EVBP + MOPP		1 (1)	
MOPP		2 (3)	
MOPP/AVB(D)		23 (29)	
Secondary chemotherapy treatment		6 (8)	
Secondary chemotherapy regimen			
DHAP		3 (4)	
MOPP		2 (3)	
MOPP/AVB		1 (1)	
Anthracycline-containing chemotherapy		70 (88)	
High-dose anthracycline–containing chemotherapy^b^		12 (15)	
Mediastinal radiotherapy		80 (100)	
Mediastinal radiotherapy boost		12 (15)	
Total mediastinal doses (Gy)		36 (36-40)	
Cardiovascular risk factors and comorbidities			
Diabetes mellitus		3 (4)	
Hypertension		13 (16)	
Hyperlipidemia		10 (13)	
Tobacco use			
Current smoker		3 (4)	
Former smoker		22 (28)	
Known coronary artery disease		0 (0)	
Hypothyroidism		25 (31)	
Hyperthyroidism		3 (4)	
Symptoms			
Chest pain		2 (3)	
Dyspnea on exertion		8 (10)	
Medications			
Angiotensin-converting enzyme inhibitor/ angiotensin receptor blocker		10 (13)	
β-blocker		4 (5)	
Calcium-channel blockers		3 (4)	
Diuretics		7 (9)	
Statins		13 (16)	
Anticoagulation			
Aspirin		5 (6)	
Coumarin derivates		2 (3)	
Laboratory results			
Estimated glomerular filtration rate (mL/min/1.73 m^2^)		90 (83-96)	
NT-proBNP (pg/ml)		93 (51-169)	

Values are mean ± SD, n (%), or median (interquartile range).

AVB(D) = Adriamycin (doxorubicin), vincristine, bleomycin (dacarbazine); BEACOPP = bleomycin, etoposide, Adriamycin (doxorubicin), cyclophosphamide, vincristine, procarbazine, prednisone; CHOP = cyclophosphamide, hydroxydaunorubicin, vincristine, prednisone; CMR = cardiovascular magnetic resonance; DHAP = dexamethasone, cytarabine, cisplatin; EBVP = epirubicin, bleomycin, vinblastine, prednisone; MOPP = mitoxantrone, vincristine, procarbazine, prednisone; NT-proBNP = N-terminal pro–B-type natriuretic peptide.

a Grades are defined according to the European Organization for Research and Treatment of Cancer risk classification for supradiaphragm stadium I and II Hodgkin lymphoma.

b High-dose anthracycline: doxorubicin ≥300 mg/m^2^, epirubicin ≥540 mg/m^2^, or mitoxantrone ≥90 mg/m^2^.

Most lymphoma survivors had a history of stage II disease (74%), and mean age at diagnosis was 27 ± 9 years. All survivors had been treated with mediastinal radiotherapy, with a median prescribed total dose of 36 Gy (interquartile range: 36-40 Gy). This local treatment was combined with systemic anthracycline chemotherapy in 70 of the survivors (88%), of whom 12 (15%) received high-dose anthracyclines. A total of 11 lymphoma survivors (14%) experienced a relapse requiring additional radiotherapy and/or anthracycline chemotherapy. The mean interval between diagnosis and CMR was 20 ± 8 years. Cardiovascular risk factors and medication are described in Table 1. Electrocardiographic and echocardiographic parameters are shown in Table 2. There was a low prevalence of electrocardiographic abnormalities. Grade II LV diastolic dysfunction and any hemodynamically significant (moderate or greater) valvular disease were found in 4 (5%) and 10 (13%) of the survivors, respectively.

**Table 2:** Electrocardiographic and Transthoracic Echocardiographic Data of (Non)-Hodgkin Lymphoma Survivors (n = 80)

Electrocardiographic characteristics	
Sinus rhythm	80 (100)
PR interval (ms)	162 ± 25
PR interval > 200 ms	4 (5)
QRS duration (ms)	97 ± 17
Left bundle branch block	1 (1)
Right bundle branch block	4 (5)
QT interval (ms)	378 ± 29
Corrected QT interval (ms)	421 ± 22
Tallest T-wave amplitude (mV)	6 ± 3
T-wave amplitude in lead aVR (mV)	3 ± 1
Left ventricular diastolic function	
Diastolic function	
Normal	46 (58)
Grade I	23 (29)
Grade II	4 (5)
Unclear	7 (9)
E wave (cm/s) (n = 75)	0.71 (0.58-0.84)
A wave (cm/s) (n = 75)	0.76 (0.55-0.91)
E/A ratio (n = 75)	0.92 (0.75-1.20)
Medial e′ (cm/s) (n = 71)	8 ± 2
E/e′ ratio (n = 69)	8 (7-10)
Systolic pulmonary artery pressure (mm Hg) (n = 39)	21 (18-26)
Left atrial volume index (mL/m^2^) (n = 61)	28 ± 6
Valvular heart disease	
Moderate or greater aortic regurgitation	3 (4)
Moderate or greater aortic stenosis	2 (3)
Moderate or greater mitral valve regurgitation	1 (1)
Moderate or greater mitral valve stenosis	3 (4)
Moderate or greater tricuspid regurgitation	1 (1)
Moderate or greater tricuspid stenosis	0 (0)
Moderate or greater pulmonary regurgitation	0 (0)
Moderate or greater pulmonary stenosis	0 (0)

Values are n (%), mean ± SD, or median (interquartile range).

Differences in CMR characteristics between lymphoma survivors and healthy control subjects are shown in Table 3 and the Central Illustration. Significantly lower LV ejection fraction (53% ± 5% vs 60% ± 5%; *P* < 0.001) and LV mass (47 ± 10 g/m^2^ vs 56 ± 8 g/m^2^; *P* < 0.001) and higher LV end-systolic volume (37 ± 8 mL/m^2^ vs 33 ± 7 mL/m^2^; *P* = 0.013) were found in lymphoma survivors. Moreover, they showed significantly lower right ventricular volumes than healthy control subjects (*P* < 0.001 for all) (Table 3), while right ventricular ejection fraction was similar between both groups (54% ± 5% vs 53% ± 4%; *P* = 0.328). GLS (−19.5% ± 2.5% vs −20.6% ± 2.0%; *P* = 0.013), GCS (−17.9% ± 2.5% vs −20.4% ± 2.2%; *P* < 0.001), and global radial strain (69% ± 15% vs 76% ± 15%; *P* = 0.018) of the LV were reduced in lymphoma survivors. Furthermore, the lateral-to-septal hemodynamic force was significantly lower in survivors (median 3.1% [interquartile range: 2.4%-3.7%] vs 3.5% [interquartile range: 2.9%-4.3%]; *P* = 0.031), while the apical-to-basal hemodynamic force was comparable between both groups (*P* = 0.58). No significant difference in right ventricular myocardial strain was found, in line with preserved right ventricular ejection fraction.

**Table 3:** Cardiovascular Magnetic Resonance Data of (Non)-Hodgkin Lymphoma Survivors Compared With Healthy Controls

	Healthy Control Subjects (n = 40)	Lymphoma Survivors (n = 80)	*P* Value
Left ventricle			
End-diastolic volume, indexed (mL/m^2^)	83 ± 12	78 ± 13	0.060
End-systolic volume, indexed (mL/m^2^)	33 ± 7	37 ± 8	0.013
Stroke volume, indexed (mL/m^2^)	50 ± 7	42 ± 7	<0.001
Ejection fraction (%)	60 ± 5	53 ± 5	<0.001
Mass, indexed (g/m^2^)	56 ± 8	47 ± 10	<0.001
Global longitudinal strain (%)	−20.6 ± 2.0	−19.5 ± 2.5	0.013
Global circumferential strain (%)	−20.4 ± 2.2	−17.9 ± 2.5	<0.001
Global radial strain (%)	76 ± 15	69 ± 15	0.018
Apical-to-basal hemodynamic forces (%)	15.2 (11.8–19.4)	16.3 (13.5–18.6)	0.58
Lateral-to-septal hemodynamic forces (%)	3.5 (2.9–4.3)	3.1 (2.4–3.7)	0.031
Right ventricle			
End-diastolic volume, indexed (mL/m^2^)	95 ± 16	78 ± 13	<0.001
End-systolic volume, indexed (mL/m^2^)	45 ± 10	36 ± 8	<0.001
Stroke volume, indexed (mL/m^2^)	50 ± 7	42 ± 7	<0.001
Ejection fraction (%)	53 ± 4	54 ± 5	0.33
Global longitudinal strain (%)^a^	−26.3 ± 3.4	−27.2 ± 4.2	0.26
Tissue characterization			
Myocardial T2 (ms)^b^	50 ± 2	50 ± 3	0.13
Native myocardial T1 (ms)	964 ± 25	980 ± 33	0.007
Hematocrit (%)^c^	41 ± 3	42 ± 3	0.33
Myocardial extracellular volume (%)^d^	29 ± 3	28 ± 3	0.24
Cell volume (mL/m^2^)^d^	39 ± 6	31 ± 7	<0.001
Presence of LGE (other than hinge point)	NA	9 (11)	NA
LGE pattern			
Subendocardial		3 (4)	
Midmyocardial		3 (4)	
Epicardial		2 (3)	
Transmural		1 (1)	
LGE quantification (% of left ventricle)		3.0 (1.9–4.1)	NA
Presence of hinge-point LGE		11 (14)	NA
Hinge-point LGE quantification (% of left ventricle)		1.7 (1.0–2.3)	NA

Values are mean ± SD, median (interquartile range), or n (%).

LGE = late gadolinium enhancement; NA = not applicable.

a Data were available for 37 of 40 healthy control subjects and 80 of 80 lymphoma survivors.

b Data were available in 40 of 40 healthy control subjects and 78 of 80 lymphoma survivors.

c Data were available in 25 of 40 healthy controls and 80 of 80 lymphoma survivors.

d Data were available in 23 of 40 healthy controls and 78 of 80 lymphoma survivors.

**Central Illustration:**
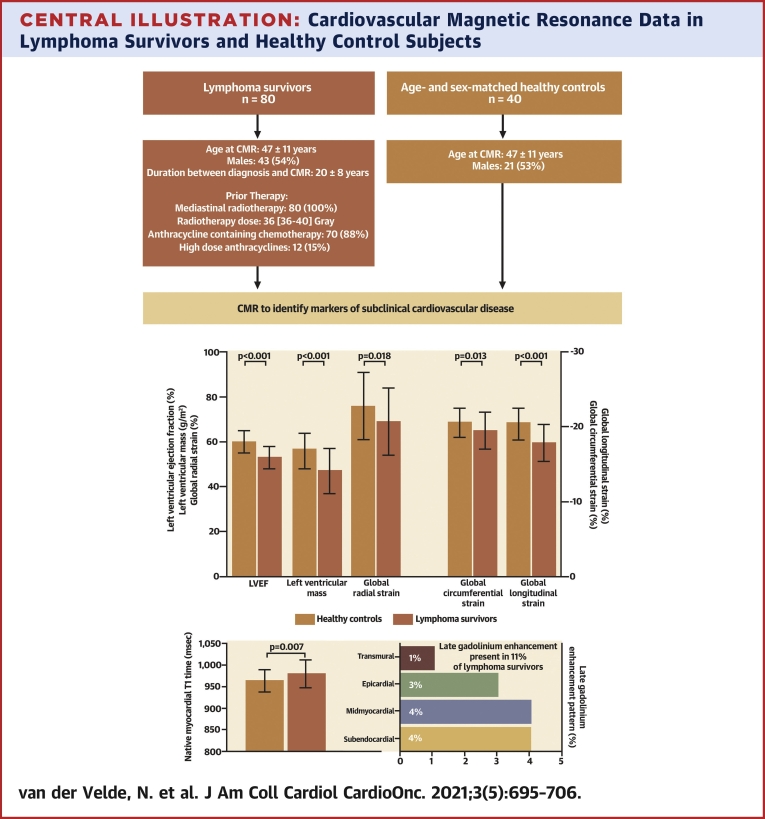
Cardiovascular Magnetic Resonance Data in Lymphoma Survivors and Healthy Control Subjects Lymphoma survivors had significantly reduced left ventricular ejection fraction, left ventricular mass, global radial strain, global longitudinal strain, and global circumferential strain compared with healthy control subjects. Native myocardial T1 was significantly higher in lymphoma survivors, and late gadolinium enhancement was detected in 11% of the survivors. LVEF = left ventricular ejection fraction.

Both groups were similar with respect to extracellular volume fraction and myocardial T2; however, statistically significant differences regarding myocardial T1 (980 ± 33 ms vs 964 ± 25 ms; *P* = 0.007) and cell volume fraction (31 ± 7 mL/m^2^ vs 39 ± 6 mL/m^2^; *P* < 0.001) were observed, with higher values in lymphoma survivors. LGE, other than at the hinge point location, was detected in 11% of survivors (median LGE quantification 3.0% [interquartile range: 1.9%-4.1%] of left ventricle). Subendocardial and transmural LGE patterns, most likely due to myocardial infarction, were present in 4 of the survivors (5%). Further evaluation by coronary computed tomographic angiography in 6 survivors revealed in 1 survivor a significant stenosis in an intermediate branch, consistent with an area of subendocardial LGE. In 11 survivors (14%), hinge-point LGE was found (median LGE quantification 1.7% [interquartile range: 1.0%-2.3%] of LV). LGE images are presented in Supplemental Figure 1.

When evaluating survivors stratified by radiotherapy dose, only a few differences were found: LV stroke volume and all right ventricular dimensions were significantly lower in survivors who received a total mediastinal radiotherapy dose >36 Gy (Supplemental Table 1). High-dose anthracyclines did not have an effect on LV dimensions, LV and right ventricular systolic function, LV mass, myocardial deformation, and tissue characterization either, although only 12 lymphoma survivors received high-dose anthracycline chemotherapy. Only a significantly lower right ventricular end-systolic volume was found in these survivors compared with the 58 survivors who received low-dose anthracyclines (Supplementa1 Table 1).

Results of the univariable and multivariable analysis for predictors of LV ejection fraction and GLS are shown in Tables 4 and 5, and predictors of GCS and LV mass are shown in Supplemental Tables 2 and 3. In our multivariable model 1, which included variables with *P* values < 0.20 in univariable analysis, total mediastinal radiotherapy dose (β = −0.22; 95% CI: −0.43 to −0.0; *P* = 0.023) and diabetes (β = −7.64; 95% CI: −13.20 to −2.08; *P* = 0.008) were found to be independent significant predictors of LV ejection fraction. Male sex (β = 1.00; 95% CI: 0.16 to 1.99; *P* = 0.046), time between diagnosis and CMR (β = 0.10; 95% CI: 0.02-0.17; *P* = 0.017), and diabetes mellitus (β = 4.49; 95% CI: 1.80-7.18; *P* = 0.001) were associated with impaired GLS. For GCS, male sex (β = 1.11; 95% CI: 0.05-2.17; *P* = 0.041) and body mass index (β = 0.13; 95% CI: 0.00-0.26; *P* = 0.046) were found to be predictors. Male sex (β = 26.68; 95% CI: 18.75-34.61; *P* < 0.001), body mass index (β = 1.39; 95% CI: 0.39-2.39; *P* = 0.007), and total mediastinal radiotherapy dose (β = −0.79; 95% CI: −1.54 to −0.05; *P* = 0.038) were associated with LV mass.

**Table 4:** Linear Regression Analysis for Predictors of Left Ventricular Ejection Fraction in Lymphoma Survivors

	Univariable Analysis			Multivariable Model 1 (*R*^2^ = 0.183)			Multivariable Model 2 (*R*^2^ = 0.193)		
	β	95% CI	*P* Value	β	95% CI	*P* Value	β	95% CI	*P* Value
Male	−1.71	−3.86 to 0.45	0.12	−1.48	−3.51 to 0.54	0.15	−1.45	−3.53 to 0.64	0.17
Age at CMR	−0.04	−0.14 to −0.06	0.40						
Duration between diagnosis and CMR	−0.08	−0.21 to 0.05	0.20				0.005	−0.13 to 0.14	0.94
Body mass index	−0.19	−0.45 to 0.06	0.13	−0.08	−0.33 to 0.17	0.52	−0.09	−0.34 to 0.16	0.49
Heart rate	0.006	−0.09 to 0.10	0.91						
Total mediastinal radiotherapy doses	−0.20	−0.40 to 0.00	0.050	−0.22	−0.43 to −0.01	0.023	−0.20	−0.41 to 0.03	0.053
High-dose anthracycline–containing chemotherapy	1.12	−1.88 to 4.21	0.45						
Total dose anthracycline-containing chemotherapy	0.005	0.00 to 0.01	0.25				0.004	−0.01 to 0.01	0.35
Diabetes mellitus	−7.73	−13.21 to −2.26	0.006	−7.64	−13.20 to −2.08	0.008	−7.84	−13.52 to −2.17	0.007
Hyperlipidemia	1.96	−1.31 to 5.23	0.24						
Hypertension	−0.09	−3.05 to 2.87	0.95						
Hypothyroidism	0.50	−1.86 to 2.85	0.68						
Hyperthyroidism	−0.84	−6.58 to 4.91	0.77						
Current or former smoker	0.80	−1.55 to 3.15	0.50						

All variables with *P* values <0.20 in the univariable linear regression were included in multivariable model 1. In multivariable model 2, the following variables irrespective of *P* value were added to the model: duration between diagnosis and CMR and total dose of anthracycline-containing chemotherapy.

**Table 5:** Linear Regression Analysis for Predictors of Global Longitudinal Strain in Lymphoma Survivors

	Univariable Analysis			Multivariable Model 1 (*R*^2^ = 0.333)			Multivariable Model 2 (*R*^2^ = 0.337)		
	β	95% CI	P Value	β	95% CI	P Value	β	95% CI	P Value
Male	1.29	0.21 to 2.37	0.020	1.00	0.16 to 1.99	0.046	1.02	0.03 to 2.01	0.044
Age at CMR	0.05	0.00 to 0.10	0.048	−0.04	−0.09 to 0.03	0.25	−0.04	−0.10 to 0.02	0.20
Duration between diagnosis and CMR	0.12	0.06 to 0.18	<0.001	0.10	0.02 to 0.17	0.017	0.10	0.02 to 0.18	0.014
Body mass index	0.12	−0.01 to 0.25	0.062	0.07	−0.05 to 0.20	0.24	0.07	−0.05 to 0.20	0.24
Heart rate	0.02	−0.03 to 0.07	0.38						
Total mediastinal radiotherapy doses	0.07	−0.03 to 0.18	0.16	0.04	−0.05 to 0.20	0.43	0.05	−0.05 to 0.14	0.37
High-dose anthracycline–containing chemotherapy	0.59	−0.97 to 2.15	0.45						
Total doses anthracycline-containing chemotherapy	0.00	−0.01 to 0.00	0.92				0.001	0.00 to 0.01	0.50
Diabetes mellitus	4.79	2.06 to 7.52	0.001	4.49	1.80 to 7.18	0.001	4.44	1.73 to 7.15	0.002
Hyperlipidemia	0.54	−1.14 to 2.23	0.52						
Hypertension	0.52	−0.99 to 2.03	0.50						
Hypothyroidism	0.93	−0.25 to 2.12	0.12	0.65	−0.48 to 1.78	0.25	0.64	−0.50 to 1.78	0.26
Hyperthyroidism	0.87	−2.06 to 3.80	0.56						
Current or former smoker	0.73	−0.47 to 1.92	0.23						

All variables with *P* values <0.20 in the univariable linear regression were included in multivariable model 1. In multivariable model 2, the following variables irrespective of *P* value were added to the model: duration between diagnosis and CMR and total dose of anthracycline-containing chemotherapy. A positive β value represents worsening of function.

In a sensitivity analysis in which anthracycline dose and time between diagnosis and CMR were also included in the model (multivariable model 2), the effect of total mediastinal radiotherapy dose for the association with LV ejection fraction was comparable with that in multivariable model 1 (β = −0.20; 95% CI: −0.41 to 0.03), although the result was no longer statistically significant (*P* = 0.053). A similar result occurred with regard to the association between total mediastinal radiotherapy dose and LV mass. Adding anthracycline dose and time between diagnosis and CMR to the models for GLS and GCS did not change the results. As diabetes mellitus appeared to be a strong predictor of LV ejection fraction and LV mass, but only 3 lymphoma survivors with diabetes were included, a sensitivity analysis was performed excluding these 3 survivors. The results did not substantially change (Supplemental Tables 4 to 7).

## Discussion

In this study, we investigated subclinical CVD in survivors of HL and NHL, treated with radiotherapy and/or anthracycline chemotherapy, without known CVD at a mean of 20 years after diagnosis. The main findings are that lymphoma survivors had significantly lower LV ejection fraction, worse myocardial strain parameters, and smaller LV mass. Furthermore, higher native myocardial T1 was found in lymphoma survivors, and LGE was present in 11%. These results indicate that lymphoma survivors are not exempt from subclinical CVD.

Cardiotoxicity can occur at different times after cancer treatment: 1) acute (<14 days); 2) early (1 week to 1 year); and 3) late (average 7 years) after the initiation of treatment. Acute onset cardiotoxicity is characterized by arrhythmias, acute coronary syndrome, myocarditis, transient LV dysfunction including acute heart failure, and electrocardiographic abnormalities, which can be reversible. However, there may also be myocardial, valvular, or coronary endothelial injury that can eventually progress to early or late onset cardiotoxicity. This can be irreversible, with a poor prognosis (1,14). Early onset cardiotoxicity can present as a dilated-hypokinetic or restrictive cardiomyopathy, which can progress to heart failure (1,4,14). Late onset cardiotoxicity can be characterized by the manifestation of valvular disease and premature or progressive coronary artery disease in addition to the early onset characteristics (1,14).

The prevalence of electrocardiographic abnormalities in our study was low and in line with a previous study in long-term lymphoma survivors (15). However, significantly higher heart rates in lymphoma survivors were found, which may indicate the presence of conduction disturbances secondary to radiotherapy-mediated injury of the conduction system. This may result in autonomic dysfunction characterized by decreased parasympathetic or increased sympathetic activity or a combination thereof (1,16). Valvular disease develops over years, with an estimated incidence of 10% in treated lymphoma survivors, and increases significantly from a total mediastinal radiotherapy dose ≥30 Gy (1,6,8,17). In contrast to other studies, we did not find a high prevalence of valvular disease (6,15,17). This can be explained by the fact that survivors with surgical interventions for valvular disease were excluded from our study and that different definitions of clinically significant valvular disease were used (6,17).

As is known from published research, LV mass may decline following anthracycline administration, with an inverse relationship between dose and LV mass (10,18). The exact pathophysiology behind this process is not fully elucidated, but it has been hypothesized that anthracyclines causes injury to the cardiomyocytes, resulting in extracellular remodeling, atrophy, or apoptosis (19). We also observed reduced LV mass and reduced cell volume with normal extracellular volume in lymphoma survivors, which might suggest cardiomyocyte atrophy. Of note, although LV mass in our lymphoma survivors was lower than in control subjects, it was still within the normal range (20). Analysis showed no significant relationship between LV mass and high-dose anthracyclines. Although it cannot be excluded that this lack of relationship was due to power, our multivariable analysis showed a significant inverse relationship between LV mass and total mediastinal radiotherapy dose. This assumption is supported by the findings of Adams et al (21), which showed significantly lower LV mass in HL survivors who had been treated with mantle-field radiotherapy. However, it is difficult to distinguish the influence of each therapy separately, as lymphoma survivors are often treated with both radiotherapy and anthracyclines. Finally, the consequences of reduced LV mass are not clear, although one might speculate that these patients are at increased risk for heart failure.

Small but significantly worse LV function and differences in volumes were found in lymphoma survivors. Only LV end-systolic volume was significantly higher in lymphoma survivors. This LV remodeling may develop because of myocardial damage from earlier treatment with radiotherapy and/or anthracyclines (1,22, 23, 24, 25). LV strain parameters were also significantly worsened in lymphoma survivors. These findings are consistent with those of previous studies that investigated strain in lymphoma survivors, with observations of significantly reduced echocardiographic GLS and GCS both early and late after treatment with chemotherapy (26,27). Although it is also difficult to determine the influence of both therapies separately here, total mediastinal radiotherapy dose was not found to be an independent predictor of GLS, and neither radiotherapy nor anthracyclines were associated with GCS. Nevertheless, we can conclude that LV function and strain parameters are markers of the detection of subclinical CVD and should be incorporated as standard parameters in clinical protocol before, during, and after cancer therapy (8).

Native myocardial T1 was significantly higher in lymphoma survivors compared with healthy control subjects. This may suggest that diffuse myocardial fibrosis is actually present in lymphoma survivors, but it may still be explained by partial volume effect due to decrease of the amount of myocardial tissue. Extracellular volume fraction, which is associated with native T1, was not increased in lymphoma survivors, although our control subjects also demonstrated higher than expected myocardial extracellular volume. However, research has shown that extracellular volume fraction has a lower discriminatory performance compared with native T1 (28). Previous studies that demonstrated elevated native myocardial T1 and/or extracellular volume fractions in patients treated with anthracyclines were either conducted at the time of anthracycline treatment or did not concern only lymphoma survivors (24,29).

Subendocardial and transmural LGE was present in 5% of lymphoma survivors, which was most likely consistent with myocardial infarction. This can be a consequence of coronary artery disease due to treatment with radiotherapy. In 6% of the survivors, the LGE pattern was midmyocardial or epicardial. No differences were found in the presence and localization of LGE between higher and lower doses of radiotherapy and/or anthracyclines. LGE at the hinge points was also prevalent in our cohort, although this is not specific for late cardiotoxicity. Hinge-point LGE is rather common in the (older) general population but also in specific populations such as athletes, making it difficult to identify the cause of this nonischemic LGE pattern.

Cardio-oncology is a relatively new field and still growing because of increased survival rates. More knowledge about subclinical CVD in long-term survivors of HL is needed for the implementation of preventive and/or therapeutic treatment in order to reduce its burden (1). Therefore, more and more research is being undertaken into risk assessment for cardiotoxicity, and its (preventive) therapeutic treatment. The first step is to perform baseline assessment of cardiovascular risk factors, alongside assessment of baseline cardiac function. It is recommended to refer high-risk patients to cardiologists for detailed cardiovascular assessment, after which any necessary preventive treatment can be initiated to minimize the risk for developing cardiotoxicity. The first studies indicated that β-blockers and angiotensin-converting enzyme inhibitors may have beneficial effects for the primary prevention of radiotherapy and/or anthracycline-induced cardiotoxicity, although more studies are necessary and long-term data are lacking (30). During and after treatment with anthracyclines, patients with cancer should be properly monitored, preferably by a specialized cardio-oncology team.

From this study we can deduce that lymphoma survivors are not exempt from subclinical CVD in comparison with healthy control subjects. This manifests mainly in changes in LV function, mass, and native myocardial T1. CMR is a suitable imaging modality for the detection of these changes, mainly because of its unique ability of tissue characterization. To the best of our knowledge, only a few studies have been performed to detect subclinical CVD using CMR in this study population (24,29). Therefore, more research is necessary to optimally manage and support these long-term survivors for cardiovascular complications. In addition, it is important to investigate the implications of changes in LV function, mass, and native T1 in relation to clinical outcomes. This will improve the identification of survivors in whom further follow-up and treatment are indicated. Furthermore, it is important to study if early medical intervention can positively influence these changes in lymphoma survivors.

### Study limitations

Although comprehensive CMR was performed, the sample size was relative small, with only 80 lymphoma survivors included. These survivors were compared with age- and sex-matched healthy control subjects. However, unmeasured confounding cannot be excluded. There is selection bias by including only less affected lymphoma survivors due to the exclusion of patients with previous surgical or percutaneous intervention for valvular or coronary artery disease. However, we still found evident signs of subclinical CVD, which underscores the severity of the problem in this relatively young study population. However, we acknowledge that we did not adjust for multiple testing, and as such our results are susceptible to type I error. Only 2 patients with NHL were included, and therefore no conclusion can be drawn regarding HL versus NHL. All consecutive patients fulfilling the inclusion criteria of the PROCARBI study were included, and the treatment of HL and mediastinal NHL is comparable, so we left these survivors in the analysis. In addition, stress perfusion CMR for the detection of ischemia was not performed, so in theory, asymptomatic coronary artery disease could have been missed. Finally, no conventional tests (ie, electrocardiography and transthoracic echocardiography) were performed in healthy control subjects.

## Conclusions

Lymphoma survivors are not exempt from CVD, which can be detected by changes in LV function and native myocardial T1 with CMR. Further longitudinal studies are needed to assess the implication of these changes in relation to clinical outcomes.

Perspectives **COMPETENCY IN MEDICAL KNOWLEDGE:** Long-term survivors of HL and mediastinal NHL lymphomas experience late adverse effects of mediastinal radiotherapy and/or anthracyclines, which lead to premature cardiovascular morbidity and mortality. Markers of CVD on CMR can detect changes in LV myocardial function and native myocardial T1. Lymphoma survivors treated with mediastinal radiation therapy and 88% received anthracyclines, had lower LV ejection fraction and LV mass compared with control subjects. LV global strain parameters were also worse. Our multivariable analysis showed that total mediastinal radiotherapy dose was significantly associated with LV ejection fraction, while male sex and duration between diagnosis and CMR were significantly associated with worsened GLS. Regular cardiac evaluation with cardiac imaging should be considered in lymphoma survivors for the detection of subclinical CVD.

**TRANSLATIONAL OUTLOOK:** Further longitudinal studies are needed to assess the implications of changes in LV function and native myocardial T1 in relation to clinical outcome.

## Funding Support and Author Disclosures

The authors have reported that they have no relationships relevant to the contents of this paper to disclose.
