# Supplementary material for: Detection of Subclinical Cardiovascular Disease by Cardiovascular Magnetic Resonance in Lymphoma Survivors
Source: JACC CardioOncol. 2021 Dec 21;3(5):695–706. doi: 10.1016/j.jaccao.2021.09.015 (PMC8702791; doi:10.1016/j.jaccao.2021.09.015)
Supplement: Supplemental Data [file mmc1.docx]

**Detection of Subclinical Cardiovascular Disease by Cardiovascular Magnetic Resonance in Lymphoma Survivors**

**Running Title:** CMR in Lymphoma Survivors

Nikki van der Velde, MD^a,b^, Cécile P.M. Janus, MD^c^, Daniel J. Bowen^a^, H. Carlijne Hassing, MD, PhD^a,b^, Isabella Kardys, MD, PhD^a^, Flora E. van Leeuwen, PhD^d^, Cynthia So-Osman, MD, PhD^e^, Remi A. Nout, MD PhD^c^, Olivier C. Manintveld, MD, PhD^a^, Alexander Hirsch, MD, PhD^a,b^

^a^Department of Cardiology, Thoraxcenter, Erasmus MC, University Medical Center Rotterdam, Rotterdam, The Netherlands;

^b^Department of Radiology and Nuclear Medicine, Erasmus MC, University Medical Center Rotterdam, Rotterdam, The Netherlands;

^c^Department of Radiation Oncology, Erasmus MC Cancer Institute, Rotterdam, The Netherlands;

^d^Department of Psychosocial Research and Epidemiology, Netherlands Cancer Institute, Amsterdam, the Netherlands;

^e^Department of Hematology, Erasmus MC, University Medical Center Rotterdam, Rotterdam, the Netherlands

**Supplemental Material**

**Supplementary methods**

*Definitions*

Hodgkin lymphoma is staged using the Ann Anbor system:

- Stage I: involvement of one lymph node region or lymphoid structure
- Stage II: involvement of two or more lymph node regions on the same side of the diaphragm
- Stage III: involvement of lymph nodes on both sides of the diaphragm
- Stage IV: involvement of extra-nodal sites other than one contiguous or proximal extra-nodal site

Hodgkin lymphoma survivors are classified as “favorable” in case of no risk factors (age <50 years + an erythrocyte sedimentation rate <50 mm without B-symptoms or an erythrocyte sedimentation rate <30 mm with B-symptoms + mediastinal tumor ratio <0.35 (=width mediastinal tumor/thorax diameter on Th5 level on a standing chest X-ray) + ≤3 affected lymph nodes regions (see figure below). Hodgkin lymphoma survivors are classified as “unfavorable” in case of the presence of risk factors (age ≥50 years + an erythrocyte sedimentation rate ≥50 mm without B-symptoms or an erythrocyte sedimentation rate ≥30 mm with B-symptoms + mediastinal tumor ratio ≥0.35 (=width mediastinal tumor/thorax diameter on Th5 level on a standing chest X-ray) + >4 affected lymph nodes regions (1, 2).

*Electrocardiogram and echocardiography*

A standard 12-lead electrocardiogram was performed in rest, and was scored for heart rate, rhythm, conduction times, T-wave amplitude, and ST-segment deviations. In case of prolongation of the QRS-complex (QRS >120ms), the type of bundle branch block was noted. T-wave amplitude was measured in the aVR lead as well as in the lead with the tallest T-wave (3).

Transthoracic echocardiography’s in lymphoma survivors were performed on a Philips Epiq 7C using a standardized acquisition protocol based on the recommendations of the American Society of Echocardiography and the European Association of Cardiovascular Imaging (4-6).

*Cardiovascular magnetic resonance*

The imaging protocol for lymphoma survivors consisted of 1) retrospectively electrocardiogram gated steady-state free precession cine imaging, 2) pre- and post-contrast T1-mapping, 3) T2-mapping, and 4) two-dimensional phase sensitive late gadolinium enhancement imaging. In healthy controls, the same imaging protocol was used with the exception of late gadolinium enhancement imaging and post-contrast T1-mapping in a subset of healthy controls.

*Steady-state free precession cine imaging*: left ventricular (LV) and right ventricular (RV) dimensions, systolic function, and LV mass were determined on the short-axis images, by manually drawing epi- and endocardial contours, without inclusion of papillary muscles and trabeculations in end-systolic and end-diastolic phase. Volumes and mass were corrected for body-surface area. Strain analyses using two-dimensional feature tracking CMR were performed. Contours were manually drawn in end-systolic and end-diastolic phase, and subsequently automatically tracked during the entire cardiac cycle, and adjusted in case of inaccurate tracking by visual assessment. Myocardial LV global longitudinal strain (GLS) was measured using all long-axis views. Endocardial RV GLS was measured on the 4-chamber view. Myocardial global circumferential strain (GCS) and myocardial global radial strain measurements were performed using a basal, mid-ventricular, and apical short-axis view. In addition, systolic and diastolic hemodynamic forces were calculated per entire heartbeat. Hemodynamic force is a new parameter that is able to detect early alterations in cardiac function by the estimation of forces using a mathematical model on conventional CMR images, directed along the longitudinal (apex-base) and transverse (inferior-anterior and septal-lateral) LV axis (7, 8). The same long-axis endocardial strain contours were used for these measurements. Typical scan parameters were slice thickness 6 mm, interslice gap 4 mm, TR/TE 4.0-4.2/1.8-1.9 ms, flip angle 65-85°, ASSET 2, field of view 340-390x240-390 mm, acquired matrix 200x280, and 30 phases per cardiac cycle.

*T1-mapping*: images for T1-mapping were obtained in a mid-ventricular short-axis slice, using a modified look-locker inverse recovery sequence with a 5(3)3 acquisition scheme pre-contrast and a 4(1)3(1)2 acquisition scheme post-contrast. Epi- and endocardial contours, whereby partial volume was taken into account, were manually drawn for the calculation of LV myocardial T1 and T2 times and extracellular and cell volume fraction. The whole myocardium in the mid-ventricular slice was included. Only in the case of artifacts, part of the myocardium was excluded. Motion correction was performed. Typical scan parameters were slice thickness 8 mm, TE/TR 1.5-1.7/3.5-3.8, flip angle 35°, ASSET 2, field of view 340-390x240-390 mm, and acquired matrix 192x140.

*T2-mapping:* the same slice location as T1-mapping was used for T2-mapping. Typical scan parameters were slice thickness 8 mm, number of echo’s 4, echo train length 24, flip angle 90°, ASSET 2, field of view 320-390x220-390 mm, and acquired matrix 224x160.

*Late gadolinium enhancement (LGE) imaging*: typical scan parameters were slice thickness 8 mm, interslice gap 2 mm, TR/TE 6.1/3.0 ms, flip angle 25°, ASSET 1.5, field of view 320-400x220-400 mm, and acquired matrix 192x160. LGE was quantified by manually drawing epicardial and endocardial contours (excluding papillary muscles) of the LV on each slice of the short-axis LGE images. Thereafter, one region of interest was drawn automatically in normal remote myocardium of the SA slice where LGE was visually most pronounced. Subsequently, hyperenhanced myocardium was automatically quantified as percentage of the LV using the 4 standard deviation thresholding technique.

**References**

1. Townsend W, Linch D. Hodgkin's lymphoma in adults. Lancet. 2012;380(9844):836-47.
2. Nagai H. Recent advances in Hodgkin lymphoma: interim PET and molecular-targeted therapy, Jpn J Clin Oncol 2015;45(2):137-45.
3. Arbel Y, Birati EY, Shapira I et al. T-wave amplitude is related to physical fitness status. Ann Noninvasive Electrocardiol 2012;17(3):214-8.
4. Lang RM, Badano LP, Mor-Avi V et al. Recommendations for Cardiac Chamber Quantification by Echocardiography in Adults: An Update from the American Society of Echocardiography and the European Association of Cardiovascular Imaging. J Am Soc Echocardiogr 2015;28:1-39.e14.
5. Nagueh SF, Smiseth OA, Appleton CP et al. Recommendations for the Evaluation of Left Ventricular Diastolic Function by Echocardiography: An Update from the American Society of Echocardiography and the European Association of Cardiovascular Imaging. J Am Soc Echocardiogr 2016;29:277-314.
6. Baumgartner H, Hung J, Bermejo J et al. Echocardiographic Assessment of Valve Stenosis: EAE/ASE Recommendations for Clinical Practice. J Am Soc Echocardiogr 2009;22:1-23.
7. Lapinskas T, Pedrizzetti G, Stoiber L et al. The Intraventricular Hemodynamic Forces Estimated Using Routine CMR Cine Images: A New Marker of the Failing Heart. JACC Cardiovasc Imaging 2019;12:377-379.
8. Faganello G, Collia D, Furlotti S et al. A new integrated approach to cardiac mechanics: reference values for normal left ventricle. Int J Cardiovasc Imaging 2020;36:2173-2185.


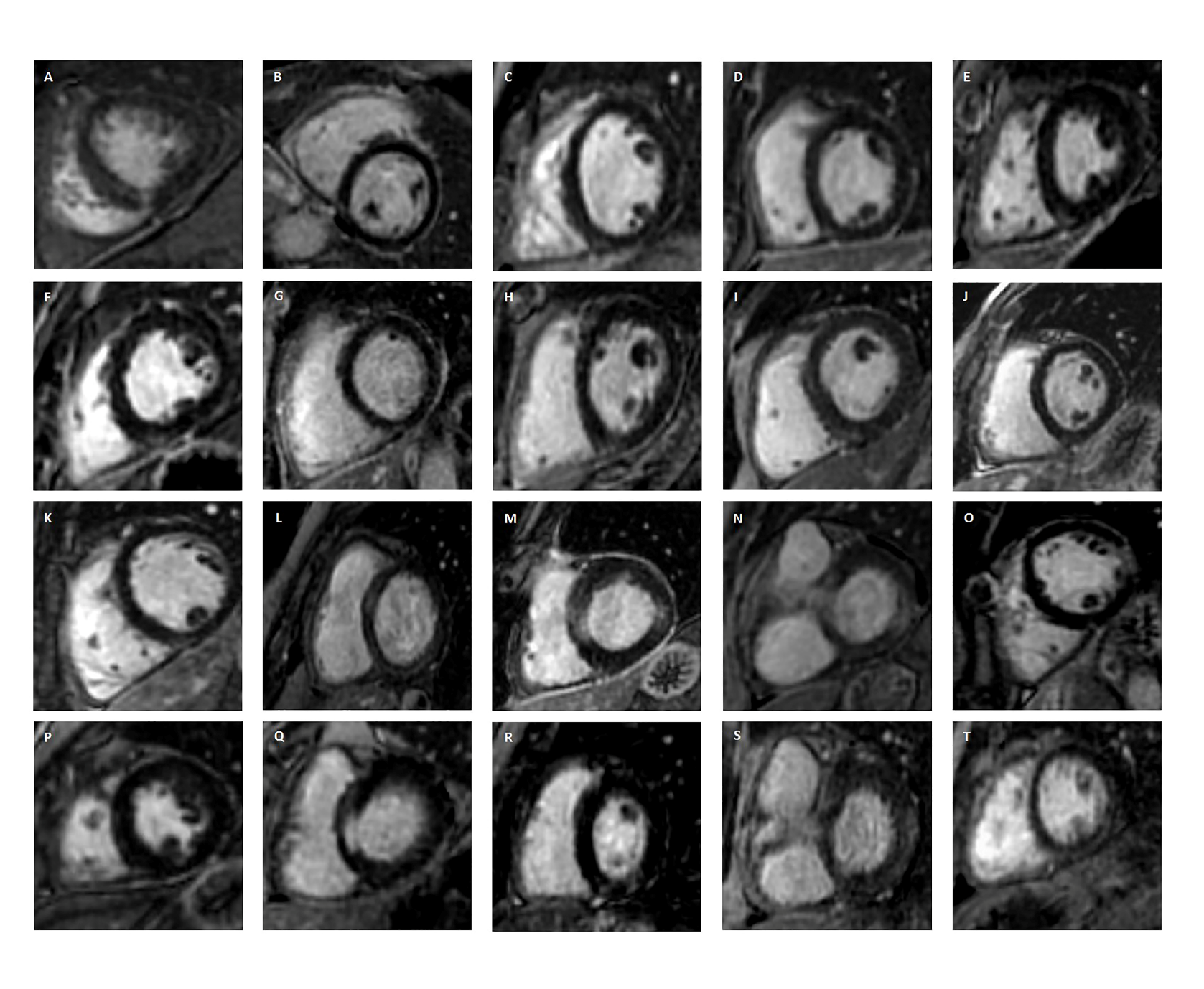
**Supplemental Figure 1: Examples of late gadolinium enhancement images in lymphoma survivors**

Short axis images of the 20 lymphoma survivors with late gadolinium enhancement (LGE) (including hinge point LGE).

**Supplemental Table 1: Cardiovascular Magnetic Resonance data of (non)-Hodgkin lymphoma survivors stratified by radiotherapy dose (median) and high dose anthracycline-containing chemotherapy**

|  | **Lymphoma survivors with radiotherapy**  **(n=80)** | | | **Lymphoma survivors with ACT**  **(n=70)** | | |
| --- | --- | --- | --- | --- | --- | --- |
|  | **Radiotherapy dose ≤36 Gray (n=42)** | **Radiotherapy dose >36 Gray (n=38)** | **p-value** | **No high dose ACT(n=58)** | **High dose ACT (n=12)** | **p-value** |
| **Left ventricle** |  |  |  |  |  |  |
| End-diastolic volume, indexed (ml/m^2^) | 81 ± 14 | 76 ± 12 | 0.072 | 79 ± 13 | 79 ± 17 | 0.942 |
| End-systolic volume, indexed (ml/m^2^) | 38 ± 6 | 36 ± 9 | 0.273 | 37 ± 6 | 37 ± 15 | 0.700 |
| Stroke volume, indexed (ml/m^2^) | 44 ± 7 | 40 ± 6 | 0.004 | 43 ± 7 | 42 ± 7 | 0.985 |
| Ejection fraction (%) | 54 ± 4 | 53 ± 6 | 0.283 | 53 ± 4 | 54 ± 7 | 0.471 |
| Mass, indexed (g/m^2^) | 48 ± 10 | 45 ± 9 | 0.122 | 47 ± 10 | 45 ± 10 | 0.551 |
| Global longitudinal strain (%) | -20.0 ± 2.3 | -18.9 ± 2.6 | 0.052 | -19.7 ± 2.4 | -19.0 ± 3.3 | 0.394 |
| Global circumferential strain (%) | -18.3 ± 2.1 | -17.4 ± 2.9 | 0.111 | -17.9 ± 2.4 | -17.5 ± 3.2 | 0.655 |
| Global radial strain (%) | 69 ± 12 | 69 ± 17 | 0.899 | 69 ± 12 | 71 ± 24 | 0.728 |
| Apex-to-base hemodynamic forces (%) | 15.6 [12.8 – 20.3] | 14.6 [11.3 – 18.8] | 0.447 | 15.2 [11.7 – 18.7] | 16.7 [10.7 – 21.9] | 0.888 |
| Lateral-to-septum hemodynamic forces (%) | 3.6 [2.9 – 4.3] | 3.5 [2.8 – 4.0] | 0.969 | 3.3 [2.8 – 4.1] | 3.8 [3.2 – 4.8] | 0.185 |
| **Right ventricle** |  |  |  |  |  |  |
| End-diastolic volume, indexed (ml/m^2^) | 82 ± 13 | 73 ± 10 | 0.001 | 80 ± 13 | 73 ± 11 | 0.079 |
| End-systolic volume, indexed (ml/m^2^) | 38 ± 8 | 34 ± 7 | 0.016 | 37 ± 8 | 31 ± 6 | 0.018 |
| Stroke volume, indexed (ml/m^2^) | 44 ± 7 | 39 ± 6 | <0.001 | 42 ± 8 | 41 ± 8 | 0.644 |
| Ejection fraction (%) | 54 ± 5 | 54 ± 6 | 0.851 | 53 ± 5 | 57 ± 5 | 0.054 |
| Global longitudinal strain (%) | -26.8 ± 3.9 | -27.5 ± 4.5 | 0.449 | -26.7 ± 4.0 | -28.9 ± 5.0 | 0.107 |
| **Tissue characterization** |  |  |  |  |  |  |
| Myocardial T2 (ms)† | 50 ± 3 | 49 ± 3 | 0.882 | 49 ± 3 | 51 ± 3 | 0.212 |
| Native myocardial T1 (ms) | 976 ± 33 | 984 ± 32 | 0.288 | 980 ± 30 | 975 ± 47 | 0.600 |
| Hematocrit (%) | 42 ± 3 | 42 ± 4 | 0.802 | 42 ± 3 | 42 ± 3 | 0.731 |
| Myocardial ECV (%)‡ | 28 ± 3 | 28 ± 3 | 0.861 | 28 ± 3 | 29 ± 3 | 0.377 |
| Cell volume (ml/m^2^)‡ | 32 ± 7 | 31 ± 6 | 0.281 | 32 ± 7 | 31 ± 6 | 0.525 |
| Presence of LGE | 5 (12%) | 4 (11%) | 0.846 | 8 (14%) | 0 (0%) | 0.335 |
| LGE pattern  Subendocardial  Midmyocardial  Epicardial  Transmural | 2 (5%)  2 (5%)  1 (5%)  0 (0%) | 1 (3%)  1 (3%)  1 (2%)  1 (2%) |  | 2 (3%)  3 (5%)  2 (3%)  1 (2%) | 0 (0%)  0 (0%)  0 (0%)  0 (0%) |  |
| LGE quantification (% of left ventricle) | 2.2 [1.9 – 6.0] | 3.1 [2.6 – 3.4] | 0.452 | 2.6 [1.8 – 4.6] | N/A | N/A |
| Presence of hinge point LGE | 4 (10%) | 7 (18%) | 0.249 | 7 (12%) | 2 (17%) | 0.664 |
| Hinge point LGE quantification (% of left ventricle) | 2.6 [2.2 – 3.1] | 1.1 [0.9 – 1.5] | 0.024 | 2.2 [1.5 – 2.7] | 0.9 [0.89/0.91] | 0.056 |

Continuous data presented as mean ± standard deviation or median [25^th^–75^th^ percentile], as appropriate. Categorical data presented as number with percentages. †Data available in 41/42 and 37/38 lymphoma survivors with radiotherapy dose ≤36 and >36 Gray; data available in 57/58 and 12/12 lymphoma survivors without and with high dose ACT, respectively. ‡Data available in 40/42 and 38/38 lymphoma survivors with radiotherapy dose ≤36 and >36 Gray; data available in 56/58 and 12/12 lymphoma survivors without and with high dose ACT, respectively.

Abbreviations: ACT = anthracycline-containing chemotherapy, ECV = extracellular volume, LGE = late gadolinium enhancement.

**Supplemental Table 2: Linear regression analysis for predictors of global circumferential strain in lymphoma survivors**

|  | **Global circumferential strain (%)** | | | | | | | | |
| --- | --- | --- | --- | --- | --- | --- | --- | --- | --- |
|  | **Univariable analysis** | | | **Multivariable model I**  **(R^2^ 0.187)** | | | **Multivariable model II**  **(R^2^ 0.196)** | | |
| **Variables** | **ß** | **95% CI** | **p-value** | **ß** | **95% CI** | **p-value** | **ß** | **95% CI** | **p-value** |
| Male sex | 1.33 | 0.25 to 2.42 | 0.017 | 1.11 | 0.05 to 2.17 | 0.041 | 1.10 | 0.04 to 2.16 | 0.043 |
| Age at CMR (years) | 0.02 | -0.03 to -0.07 | 0.39 |  |  |  |  |  |  |
| Duration between diagnosis and CMR (years) | 0.07 | 0.00 to 0.13 | 0.047 | 0.04 | -0.03 to 0.10 | 0.25 | 0.03 | -0.03 to 0.10 | 0.33 |
| Body mass index (kg/m^2^) | 0.17 | 0.04 to 0.30 | 0.009 | 0.13 | 0.00 to 0.26 | 0.046 | 0.13 | 0.01 to 0.26 | 0.041 |
| Heart rate (beats/min) | 0.003 | -0.05 to 0.05 | 0.90 |  |  |  |  |  |  |
| Total mediastinal radiotherapy doses (Gray) | 0.03 | -0.07 to 0.14 | 0.55 |  |  |  |  |  |  |
| High dose anthracycline-containing chemotherapy | 0.37 | -1.20 to 1.94 | 0.64 |  |  |  |  |  |  |
| Total doses anthracycline-containing chemotherapy (mg/m^2^) | -0.002 | -0.01 to 0.00 | 0.40 |  |  |  | -0.002 | -0.01 to 0.00 | 0.36 |
| Diabetes mellitus | 3.14 | 0.27 to 6.01 | 0.032 | 1.95 | -0.90 to 4.80 | 0.18 | 2.10 | -0.77 to 4.97 | 0.15 |
| Hyperlipidemia | 0.64 | -1.05 to 2.34 | 0.45 |  |  |  |  |  |  |
| Hypertension | 0.37 | -1.15 to 1.89 | 0.63 |  |  |  |  |  |  |
| Hypothyroidism | -0.04 | -1.25 to 1.17 | 0.95 |  |  |  |  |  |  |
| Hyperthyroidism | 0.94 | -2.01 to 3.89 | 0.53 |  |  |  |  |  |  |
| Current or former smoker | -0.26 | -1.47 to 0.96 | 0.68 |  |  |  |  |  |  |

All variables with p-value of <0.200 in the univariable linear regression were included in multivariable analysis I. In multivariable analysis II the following variables irrespective of the p-value were added to the model: duration between diagnosis and CMR and total doses of anthracycline-containing chemotherapy.

A positive ß represents a worsening of function.

Abbreviations: CI = confidence interval, CMR = cardiovascular magnetic resonance.

**Supplemental Table 3: Linear regression analysis for predictors of left ventricular mass in lymphoma survivors**

|  | **Left ventricular mass (mg/m^2^)** | | | | | | | | |
| --- | --- | --- | --- | --- | --- | --- | --- | --- | --- |
|  | **Univariable analysis** | | | **Multivariable model I**  **(R^2^ 0.541)** | | | **Multivariable model II**  **(R^2^ 0.554)** | | |
| **Variables** | **ß** | **95% CI** | **p-value** | **ß** | **95% CI** | **p-value** | **ß** | **95% CI** | **p-value** |
| Male sex | 29.00 | 20.74 to 37.26 | <0.001 | 26.68 | 18.75 to 34.61 | <0.001 | 27.59 | 19.51 to 35.67 | <0.001 |
| Age at CMR (years) | 0.65 | 0.19 1.11 | 0.006 | 0.11 | -0.28 to 0.50 | 0.58 | 0.32 | -0.16 to 0.82 | 0.19 |
| Duration between diagnosis and CMR | 0.17 | -0.45 to 0.79 | 0.59 |  |  |  | -0.46 | -1.10 to 0.18 | 0.16 |
| Body mass index (kg/m^2^) | 1.80 | 0.63 to 2.97 | 0.003 | 1.39 | 0.39 to 2.39 | 0.007 | 1.28 | 0.27 to 2.29 | 0.014 |
| Heart rate (beats/min) | -0.41 | -0.87 to 0.06 | 0.083 | -0.19 | -0.55 to 0.17 | 0.30 | -0.09 | -0.48 to 0.30 | 0.65 |
| Total mediastinal radiotherapy doses (Gray) | -0.85 | -1.82 to 0.12 | 0.086 | -0.79 | -1.54 to -0.05 | 0.038 | -0.71 | -1.48 to 0.06 | 0.072 |
| High dose anthracycline-containing chemotherapy | -2.04 | -16.74 to 12.66 | 0.78 |  |  |  |  |  |  |
| Total doses anthracycline-containing chemotherapy (mg/m^2^) | 0.11 | -0.03 to 0.05 | 0.59 |  |  |  | -0.01 | -0.04 to 0.03 | 0.68 |
| Diabetes mellitus | 26.90 | -0.08 to 53.88 | 0.051 | 9.25 | -12.49 to 30.99 | 0.40 | 9.11 | -12.64 to 30.86 | 0.41 |
| Hyperlipidemia | 15.04 | -0.48 to 30.56 | 0.057 | 4.29 | -8.36 to 16.94 | 0.50 | 4.63 | -8.05 to 17.30 | 0.47 |
| Hypertension | 2.46 | -11.77 to 16.69 | 0.73 |  |  |  |  |  |  |
| Hypothyroidism | 1.06 | -10.27 to 12.39 | 0.85 |  |  |  |  |  |  |
| Hyperthyroidism | 7.03 | -20.58 to 34.63 | 0.61 |  |  |  |  |  |  |
| Current or former smoker | 8.74 | -2.42 to 19.90 | 0.12 | 1.80 | -7.01 to 10.60 | 0.69 | 2.17 | -6.93 to 11.28 | 0.64 |

All variables with p-value of <0.200 in the univariable linear regression were included in multivariable analysis I. In multivariable analysis II the following variables irrespective of the p-value were added to the model: duration between diagnosis and CMR and total doses of anthracycline-containing chemotherapy.

Abbreviations: CI = confidence interval, CMR = cardiovascular magnetic resonance.

**Supplemental Table 4: Linear regression analysis for predictors of left ventricular ejection fraction in lymphoma survivors without diabetes mellitus (n=77)**

|  | **Left ventricular ejection fraction (%)** | | | | | | | | |
| --- | --- | --- | --- | --- | --- | --- | --- | --- | --- |
|  | **Univariable analysis** | | | **Multivariable analysis I**  **(R^2^ 0.071)** | | | **Multivariable analysis II**  **(R^2^ 0.079)** | | |
| **Variables** | **ß** | **95% CI** | **p-value** | **ß** | **95% CI** | **p-value** | **ß** | **95% CI** | **p-value** |
| Male sex | -1.23 | -3.32 to 0.87 | 0.25 |  |  |  |  |  |  |
| Age at CMR (years) | 0.003 | -0.10 to 0.10 | 0.95 |  |  |  |  |  |  |
| Duration between diagnosis and CMR (years) | -0.07 | -0.19 to 0.06 | 0.30 |  |  |  | -0.04 | -0.17 to 0.09 | 0.57 |
| Body mass index (kg/m^2^) | -0.09 | -0.35 to 0.16 | 0.48 |  |  |  |  |  |  |
| Heart rate (beats/min) | 0.01 | -0.08 to 0.11 | 0.77 |  |  |  |  |  |  |
| Total mediastinal radiotherapy doses (Gray) | -0.20 | -0.39 to 0.00 | 0.052 | -0.17 | -0.37 to 0.03 | 0.092 | -0.15 | -0.36 to 0.07 | 0.17 |
| High dose anthracycline-containing chemotherapy | 0.83 | -2.07 to 3.73 | 0.57 |  |  |  |  |  |  |
| Total doses anthracycline-containing chemotherapy (mg/m^2^) | 0.005 | 0.00 to 0.01 | 0.22 |  |  |  | 0.002 | -0.01 to 0.01 | 0.68 |
| Hyperlipidemia | 1.83 | -1.43 to 5.08 | 0.27 |  |  |  |  |  |  |
| Hypertension | 0.70 | -2.21 to 3.60 | 0.64 |  |  |  |  |  |  |
| Hypothyroidism | 0.08 | -2.18 to 2.33 | 0.95 |  |  |  |  |  |  |
| Hyperthyroidism | -1.14 | -6.59 to 4.31 | 0.68 |  |  |  |  |  |  |
| Current or former smoker | 1.89 | -0.41 to 4.18 | 0.11 | 1.53 | -0.78 to 3.83 | 0.19 | 1.44 | -0.98 to 3.87 | 0.24 |

All variables with p-value of <0.200 in the univariable linear regression were included in multivariable analysis I. In multivariable analysis II the following variables irrespective of the p-value were added to the model: duration between diagnosis and CMR and total doses of anthracycline-containing chemotherapy.

Abbreviations: CI = confidence interval, CMR = cardiovascular magnetic resonance.

**Supplemental Table 5: Linear regression analysis for predictors of global longitudinal strain in lymphoma survivors without diabetes mellitus (n=77)**

|  | **Global longitudinal strain (%)** | | | | | | | | |
| --- | --- | --- | --- | --- | --- | --- | --- | --- | --- |
|  | **Univariable analysis** | | | **Multivariable model I**  **(R^2^ 0.204)** | | | **Multivariable model II**  **(R^2^ 0.206)** | | |
| **Variables** | **ß** | **95% CI** | **p-value** | **ß** | **95% CI** | **p-value** | **ß** | **95% CI** | **p-value** |
| Male sex | 1.18 | 0.13 to 2.22 | 0.028 | 0.92 | 0.10 to 1.94 | 0.075 | 0.92 | 0.10 to 1.94 | 0.076 |
| Age at CMR (years) | 0.03 | -0.02 to 0.08 | 0.26 |  |  |  |  |  |  |
| Duration between diagnosis and CMR (years) | 0.11 | 0.05 to 0.17 | 0.001 | 0.07 | 0.01 to 0.14 | 0.025 | 0.08 | 0.01 to 0.14 | 0.023 |
| Body mass index (kg/m^2^) | 0.07 | -0.06 to 0.19 | 0.32 |  |  |  |  |  |  |
| Heart rate (beats/min) | 0.02 | -0.03 to 0.06 | 0.49 |  |  |  |  |  |  |
| Total mediastinal radiotherapy doses (Gray) | 0.09 | -0.01 to 0.19 | 0.085 | 0.04 | -0.05 to 0.14 | 0.40 | 0.05 | -0.06 to 0.15 | 0.36 |
| High dose anthracycline-containing chemotherapy | 0.81 | -0.67 to 2.28 | 0.28 |  |  |  |  |  |  |
| Total doses anthracycline-containing chemotherapy (mg/m^2^) | 0.00 | -0.01 to 0.00 | 0.71 |  |  |  | 0.001 | 0.00 to 0.01 | 0.64 |
| Hyperlipidemia | 0.37 | -1.31 to 2.04 | 0.67 |  |  |  |  |  |  |
| Hypertension | 0.31 | -1.17 to 1.80 | 0.68 |  |  |  |  |  |  |
| Hypothyroidism | 1.22 | 0.10 to 2.33 | 0.033 | 0.68 | -0.45 to 1.81 | 0.23 | 0.68 | -0.45 to 1.82 | 0.23 |
| Hyperthyroidism | 1.06 | -1.71 to 3.83 | 0.45 |  |  |  |  |  |  |
| Current or former smoker | 0.17 | -1.02 to 1.36 | 0.78 |  |  |  |  |  |  |

All variables with p-value of <0.200 in the univariable linear regression were included in multivariable analysis I. In multivariable analysis II the following variables irrespective of the p-value were added to the model: duration between diagnosis and CMR and total doses of anthracycline-containing chemotherapy.

A positive ß represents a worsening of function.

Abbreviations: CI = confidence interval, CMR = cardiovascular magnetic resonance.

**Supplemental Table 6: Linear regression analysis for predictors of global circumferential strain in lymphoma survivors without diabetes mellitus (n=77)**

|  | **Global circumferential strain (%)** | | | | | | | | |
| --- | --- | --- | --- | --- | --- | --- | --- | --- | --- |
|  | **Univariable analysis** | | | **Multivariable model I**  **(R^2^ 0.130)** | | | **Multivariable model II**  **(R^2^ 0.138)** | | |
| **Variables** | **ß** | **95% CI** | **p-value** | **ß** | **95% CI** | **p-value** | **ß** | **95% CI** | **p-value** |
| Male sex | 1.19 | 0.11 to 2.28 | 0.032 | 1.01 | 0.07 to 2.10 | 0.067 | 1.02 | -0.07 to 2.11 | 0.066 |
| Age at CMR (years) | 0.01 | -0.05 to 0.06 | 0.81 |  |  |  |  |  |  |
| Duration between diagnosis and CMR (years) | 0.06 | 0.00 to 0.13 | 0.060 | 0.04 | -0.02 to 0.11 | 0.20 | 0.04 | -0.03 to 0.10 | 0.26 |
| Body mass index (kg/m^2^) | 0.13 | 0.00 to 0.27 | 0.046 | 0.12 | -0.01 to 0.25 | 0.061 | 0.13 | 0.00 to 0.26 | 0.056 |
| Heart rate (beats/min) | -0.003 | -0.05 to 0.05 | 0.91 |  |  |  |  |  |  |
| Total mediastinal radiotherapy doses (Gray) | 0.03 | -0.08 to 0.14 | 0.50 |  |  |  |  |  |  |
| High dose anthracycline-containing chemotherapy | 0.51 | -1.02 to 2.05 | 0.51 |  |  |  |  |  |  |
| Total doses anthracycline-containing chemotherapy (mg/m^2^) | -0.002 | -0.01 to 0.00 | 0.35 |  |  |  | -0.002 | -0.01 to 0.00 | 0.44 |
| Hyperlipidemia | 0.76 | -0.97 to 2.49 | 0.38 |  |  |  |  |  |  |
| Hypertension | 0.29 | -1.25 to 1.83 | 0.71 |  |  |  |  |  |  |
| Hypothyroidism | 0.13 | -1.06 to 1.33 | 0.82 |  |  |  |  |  |  |
| Hyperthyroidism | 1.06 | -1.82 to 3.94 | 0.46 |  |  |  |  |  |  |
| Current or former smoker | -0.69 | -1.92 to 0.54 | 0.27 |  |  |  |  |  |  |

All variables with p-value of <0.200 in the univariable linear regression were included in multivariable analysis I. In multivariable analysis II the following variables irrespective of the p-value were added to the model: duration between diagnosis and CMR and total doses of anthracycline-containing chemotherapy.

A positive ß represents a worsening of function

Abbreviations: CI = confidence interval, CMR = cardiovascular magnetic resonance.

**Supplemental Table 7: Linear regression analysis for predictors of left ventricular mass in lymphoma survivors without diabetes mellitus (n=77)**

|  | **Left ventricular mass (mg/m^2^)** | | | | | | | | |
| --- | --- | --- | --- | --- | --- | --- | --- | --- | --- |
|  | **Univariable analysis** | | | **Multivariable model I**  **(R^2^ 0.529)** | | | **Multivariable model II**  **(R^2^ 0.554)** | | |
| **Variables** | **ß** | **95% CI** | **p-value** | **ß** | **95% CI** | **p-value** | **ß** | **95% CI** | **p-value** |
| Male sex | 29.82 | 21.66 to 37.97 | <0.001 | 28.15 | 20.09 to 36.21 | <0.001 | 29.99 | 21.79 to 38.18 | <0.001 |
| Age at CMR (years) | 0.57 | 0.82 to 1.05 | 0.023 | 0.12 | -0.26 to 0.51 | 0.53 | 0.44 | -0.06 to 0.93 | 0.083 |
| Duration between diagnosis and CMR | 0.07 | -0.56 to 0.70 | 0.83 |  |  |  | -0.64 | -1.30 to 0.01 | 0.054 |
| Body mass index (kg/m^2^) | 1.63 | 0.40 to 2.86 | 0.010 | 1.51 | 0.51 to 2.51 | 0.004 | 1.41 | 0.41 to 2.40 | 0.006 |
| Heart rate (beats/min) | -0.43 | -0.89 to 0.04 | 0.069 | -0.15 | -0.52 to 0.21 | 0.40 | 0.001 | -0.39 to 0.39 | 0.99 |
| Total mediastinal radiotherapy doses (Gray) | -0.76 | -1.77 to 0.25 | 0.14 | -0.71 | -1.45 to 0.04 | 0.062 | -0.55 | -1.32 to 0.22 | 0.16 |
| High dose anthracycline-containing chemotherapy | -0.86 | -15.52 to 13.80 | 0.91 |  |  |  |  |  |  |
| Total doses anthracycline-containing chemotherapy (mg/m^2^) | 0.01 | -0.04 to 0.05 | 0.76 |  |  |  | -0.01 | -0.04 to 0.02 | 0.54 |
| Hyperlipidemia | 13.78 | -2.46 to 30.03 | 0.095 | 0.57 | -12.58 to 13.72 | 0.93 | -0.39 | -13.42 to 12.63 | 0.95 |
| Hypertension | 0.62 | -14.03 to 15.28 | 0.93 |  |  |  |  |  |  |
| Hypothyroidism | 2.58 | -8.76 to 13.92 | 0.65 |  |  |  |  |  |  |
| Hyperthyroidism | 8.09 | -19.33 to 35.50 | 0.56 |  |  |  |  |  |  |
| Current or former smoker | 6.03 | -5.66 to 17.72 | 0.31 |  |  |  |  |  |  |

All variables with p-value of <0.200 in the univariable linear regression were included in multivariable analysis I. In multivariable analysis II the following variables irrespective of the p-value were added to the model: duration between diagnosis and CMR and total doses of anthracycline-containing chemotherapy.

Abbreviations: CI = confidence interval, CMR = cardiovascular magnetic resonance.
